# Neurobiological and Psychological Maintenance Mechanisms Associated with Anticipatory Reward in Bulimia Nervosa

**DOI:** 10.20900/jpbs.20210005

**Published:** 2021-04-08

**Authors:** Carol B. Peterson, Ann F. Haynos, Bryon A. Mueller, Ross D. Crosby, Stephen A. Wonderlich, Lisa M. Anderson, Carolyn M. Pearson, Molly Fennig, Erin Gallagher, Kathryn R. Cullen

**Affiliations:** 1 Department of Psychiatry and Behavioral Sciences, University of Minnesota, Minneapolis, MN 55454, USA; 2 Sanford Research, Fargo, ND 58122 USA; 3 Carter Psychology, Indianapolis, IN 46240, USA

**Keywords:** bulimia nervosa, fMRI, EMA

## Abstract

The purpose of this investigation is to identify the anticipatory reward mechanisms that maintain binge eating and purging in bulimia nervosa. Emerging data indicate the importance of reward and anticipatory processes as maintenance mechanisms of bulimia nervosa that can be targeted in treatment. The proposed research will identify neurobiological and psychological anticipatory mechanisms of binge eating and purging using functional magnetic resonance imaging (fMRI), and ecological momentary assessment (EMA) in the natural environment. In this investigation, 60 adults (30 with bulimia nervosa and 30 matched comparison participants) will undergo negative and positive mood inductions followed by an fMRI food selection task (and a comparison shopping task) to examine neurobiological and affective responses to food and non-food reward anticipation. Participants with bulimia nervosa will complete two weeks of EMA examining real-time affect changes in relation to the anticipation of binge eating and purging. These methods will facilitate rigorous assessment of the links between neurobiological (fMRI) and naturalistic (EMA) data in anticipatory reward processes. Findings from this investigation will inform the conceptualization and treatment of bulimia nervosa by identifying the role of reward anticipation in symptom maintenance, providing a crucial framework for targeting these anticipatory processes in existing and novel interventions.

## INTRODUCTION

Bulimia nervosa (BN) is an eating disorder characterized by recurrent episodes of binge eating and purging along with negative self-evaluation of shape and weight. With prevalence rates of 1–3% [1], BN is associated with significant medical and psychiatric comorbidity including mood, anxiety, and personality disorders [2]. Although evidence-based interventions including cognitive-behavioral therapy (CBT) have been developed, BN often persists in spite of treatment [2–4], indicating ineffectively targeted maintenance mechanisms. Treatment outcome data suggest that only 30–45% of adults who receive treatment for BN exhibit prolonged remission [5]. Further, BN is often characterized by a worsening course in which symptom severity increases with duration of illness [6] Existing BN treatments, including CBT, typically focus on immediate antecedents and consequences of bulimic behavior. However, it is possible that the reward derived from these behaviors may occur earlier, during the *anticipation* of binge eating and purging. Determining the role of reward anticipation in BN will facilitate the application of novel interventions that more precisely target these maintenance mechanisms.

Current research suggests that BN results from a complex combination of genetic susceptibility and environmental contributors including adverse life events and sociocultural influences, along with predisposing, precipitating, and maintaining factors [2]. Longitudinal data indicate that weight and shape concerns, the internalization of the thin ideal, dieting, and body dissatisfaction among youth are predictive of BN behaviors [7], as well as relational and familial factors [8]. Adverse life events including trauma are also associated with BN [9]. Accumulating evidence has demonstrated the association between BN and phenotypic features including behavioral inhibition and disinhibition, personality traits (perfectionism, impulsivity, negative urgency), neurocognitive processes (attention biases), and neurobiological responses (e.g., activation in reward circuitry) [2]. Empirical findings are consistent with the behavioral susceptibility theory, suggesting that genetic vulnerability influences the responsiveness to food cues, appetite dysregulation, and reward learning (which are associated with functional connectivity and brain circuits associated with reward, described below) [2]. Research also supports the critical role of emotion as a longitudinal risk factor [7] and a precipitant of BN behaviors, as well as the likelihood that negative emotion enhances the reward value of food-related stimuli [2,10].

Ecological momentary assessment (EMA) research, which measures symptoms under real-time and real-world conditions, has also supported an affect regulation model to explain the rewarding nature of bulimic symptoms [11–13]. This research demonstrates that, at a group level, the occurrence of binge eating and purging is precipitated by decreasing positive affect and increasing negative affect, which, respectively, increase and decline after the occurrence of binge-eating and purging behaviors. As a result, binge eating and purging may be negatively reinforced by the reduction in the experience of negative affect and positively reinforced by increases in the experience of positive affect. Laboratory research has similarly established the temporal link between affect and bulimic behaviors as well as self-reported planning of binge-eating and purging episodes [14–16]. Emerging neurobiological data parallel findings from EMA and laboratory studies to support the role of affective processing in binge eating and purging among individuals with BN, particularly in two neural circuits. The first is the striatal approach network, which involves dopaminergic projections from the ventral tegmentum and the substantia nigra to striatal regions including nucleus accumbens, caudate, and putamen, which in turn receive regulatory input from frontal regions (e.g., orbitofrontal and ventromedial prefrontal cortex). Although multiple parallel loops within this overall system have been described [12], the ventral parts of the striatum (e.g., nucleus accumbens) are generally implicated in positive affect as well as immediate reward receipt and more dorsal portions of the striatum (e.g., caudate, putamen) are involved in cognition and motor control enacting responses informed by a history of reward learning [17]. The second network, the limbic threat network, underlies the expression and regulation of negative affect [18] and includes structures such as the amygdala, hippocampus, and insula that underlie negative emotion, while associated frontal regions (e.g., dorsolateral prefrontal cortex, anterior cingulate cortex) serve a regulatory function [18].

Individuals with BN have demonstrated enhanced reactivity in brain regions associated with the striatal approach system (e.g., nucleus accumbens, caudate, orbitofrontal cortex) and decreased reactivity in regions associated with the limbic threat system (e.g., amygdala) [17–22] during exposure to food cues, further supporting the premise that aspects of binge eating (and, potentially, purging) are associated with the processing of desirable affective states. Because binge eating and purging as well as related cues have been shown to be associated with positive reinforcement by increases in positive affect (and activity in the approach network) and negative reinforcement through reduction of negative affect (and decreased activity in the threat network), evidence-based behavioral treatments of BN have generally focused on immediate precipitants and consequences of these behaviors [23]. However, given that the majority of individuals with BN (~75%) plan some or most of their binge-eating and purging episodes [16,24], the rewarding effects of these behaviors may also occur long before the occurrence of the behavior during the distal anticipation stage, which is not emphasized in current models of BN. Although reward disturbances associated with psychopathology have often been understood in terms of generalized deficits and surfeits, more recent models recognize that the reward processes that promote symptom expression are not homogenous within psychiatric populations [25,26]. Therefore, more comprehensive theories that parse different aspects of reward processing have been encouraged to develop more accurate models of psychiatric disorders [27]. The distinction between the mechanisms of reward anticipation and reward consumption has emerged as especially important, with data indicating that disturbances in these different processes characterize different forms of psychopathology [21] and stages of illness [28]. These anticipatory mechanisms are reflected in patterns of brain activity, with individuals with addictive disorders demonstrating greater striatal activity coupled with decreased striatal connectivity to prefrontal structures during reward anticipation [29]. These data parallel animal models that indicate that dopamine release shifts temporally from reward receipt to anticipation over repeated negative or positive reinforcement trials [30,26]. In this context, reward anticipation may be an important but neglected mechanism in the conceptualization of BN, which may be particularly relevant during more chronic, treatment resistant stages of the disorder. Although existing maintenance models of BN have begun to address the role of anticipatory reward, these models have not sufficiently examined the potential reward mechanisms associated with more distal anticipation and planning. Understanding anticipatory mechanisms in BN is critical for identifying treatment targets for those who do not respond to standard treatments like CBT, which typically focus on more immediate behavioral precipitants. Along these lines, novel treatments are emerging that target anticipatory reward separately from consummatory reward mechanisms associated with eating behavior [31].

In sum, despite (a) the role of low positive affect and high negative affect as precipitants of binge eating and purging, (b) the role of post-behavioral increases in positive affect and decreases in negative affect (as well as parallel neurobiological processes) as reward processes promoting binge eating and purging, (c) robust clinical data supporting the frequency of planned binge-eating and purging episodes in BN, and (d) emerging data demonstrating the importance of reward anticipation in psychiatric disorders, the psychological and neurobiological impact of binge-eating and purging anticipation is unknown. The purpose of this study is to investigate the psychological and neurobiological aspects of reward anticipation in BN from the theoretical lens of behavioral susceptibility and reward learning. Identifying these reward processes will expand treatment targets to the anticipatory mechanisms of BN.

This investigation will use fMRI and EMA to examine anticipatory reward among adults with BN (*n* = 30) and comparison participants (*n* = 30). Specifically, fMRI procedures will include two tasks: a food choice task and a comparison (non-food related) shopping task. Affective and neurobiological responses to both tasks following negative affect and positive affect induction will be compared within participant groups with duration of illness a moderator for the BN group. The BN group will also complete a two-week EMA protocol to investigate planning and anticipation of binge-eating/purging behavior as well as affect in the naturalistic environment (because the comparison participants will have no history of binge eating, they will not complete these EMA procedures). EMA and fMRI data will be examined to determine the relationship between self-reported anticipation of binge eating and purging in the natural environment and neurobiological responses on the fMRI food choice task. The specific aims and hypotheses of the investigation are described in the following section.

## GRANT AIMS

### Aim 1. Identity the Neural and Affective Reward Correlates of Anticipating Binge Eating and Purging in BN

#### Hypothesis 1.1.

We hypothesize that following negative mood induction, participants with BN will show lower levels of self-reported negative affect, lower levels of activation in regions of the limbic threat network (e.g., amygdala, insula), and greater frontolimbic connectivity during an fMRI food choice task compared to the shopping task. We hypothesize that in contrast, healthy comparison participants will show no differentiation in these responses between tasks after a negative mood induction.

#### Hypothesis 1.2.

We hypothesize that following positive mood induction, participants with BN will show higher levels of self-reported positive affect, higher levels of activation in regions of the striatal approach network (e.g., nucleus accumbens, caudate), and lower frontostriatal connectivity during an fMRI food choice task compared to the shopping task. We hypothesize that in contrast, healthy comparison participants will show no differentiation in these responses between tasks after positive mood induction.

#### Hypothesis 1.3.

We hypothesize that for participants with BN, illness duration will moderate responses to an fMRI food choice task. Specifically, we predict that longer illness duration will be associated with: (a) less negative affect, less activation of the limbic threat network, and more frontolimbic connectivity following the negative mood induction; and (b) more positive affect, more activation of striatal approach regions, and less frontostriatal connectivity following the positive mood induction.

### Aim 2. Determine the Associations between Neurobiological, Behavioral, and Self-Reported Reward Indices Responses to Binge-Eating and Purging Behavior Anticipation in the Natural Environment

#### Hypothesis 2.1.

We hypothesize that the magnitude of EMA negative affect reduction with binge-eating and purging behavior among individuals with BN will be associated with the magnitude of limbic threat network activity and frontolimbic connectivity observed during an fMRI food choice task in the negative mood induction condition.

#### Hypothesis 2.2.

We hypothesize that the magnitude of EMA positive affect increase with binge- eating and purging behavior among individuals with BN will be associated with the magnitude of striatal approach network activity and frontostriatal connectivity during an fMRI food choice task in the positive mood induction condition.

#### Hypothesis 2.3.

We hypothesize that greater EMA b inge-eating and purging frequency will correlate with greater magnitude of reductions in negative affect and increases in positive affect associated with binge-eating and purging anticipation as measured by EMA. We also hypothesize that greater EMA binge-eating and purging frequency will also correlate with fMRI measures of limbic threat and striatal approach network activity, as well as connectivity among individuals with BN.

## INNOVATION

(1) This study is the first to propose a novel approach to establish the role of anticipatory reward mechanisms in a maintenance model of BN and to examine reward anticipation independently from reward consumption, which holds promise for identifying important novel targets for the treatment of BN.
(2) This study administers an established food choice task during fMRI to identify the extent to which the anticipation of binge-eating and purging episodes impacts reactivity and connectivity of two key neural circuits (threat and approach networks). Because there is little research on circuit connectivity in response to disorder-relevant contexts in BN, this study contributes added complexity to neurobiological models of binge eating and purging.
(3) The combination of fMRI with EMA is an emerging novel approach in psychiatric research [19,32–34] to examine the relations between neuroimaging data and naturalistic behavior and affect in the “real world”. Examining the link between self-reported affect and the neural processes associated with anticipating binge eating and purging in the scanner along with EMA is an innovative approach to establish the ecological validity of fMRI findings. This study is also the first to examine anticipation of binge eating and purging using EMA.
(4) This study provides a potential model for understanding the role of illness duration in BN by examining the extent to which reward anticipation strengthens over the course of the illness.

## PRELIMINARY WORK

### Neurobiological Changes in Reward and Emotion Regions Are Associated with Binge-Eating and Purging Anticipation in BN

Our preliminary data suggest that the anticipation of binge eating directly impacts brain regions associated with emotion and reward in BN. This pilot study [24] included nine females (mean age = 20.22, SD = 2.39), six with BN and three with no eating disorder. In the scanner, following negative mood induction, participants were shown pictures of palatable food and were instructed to plan a binge-eating episode by selecting pictures of foods that they were told that they could eat in private following the scan. Participants completed a parallel “neutral” task in which they were shown pictures of furniture that they could select to furnish an apartment. Compared to the non-BN participants, the BN group showed lower activation across the bilateral amygdala and greater activation across the bilateral caudate during the binge-eating planning compared to the furniture selection task. These findings suggest that binge eating in BN may be rewarded through altered neurobiological responses during the anticipated occurrence of binge eating and purging. However, further data are needed to examine circuit-level alterations during binge-eating and purging episode anticipation.

### EMA-Measured Changes in Negative Affect and Positive Affect Contribute to Binge-Eating and Purging Maintenance in BN and Other Eating Disorders

Using EMA, our collaborative research team has demonstrated that the trajectories of increases in negative affect and reductions in positive affect that occur prior to the onset of binge eating (and purging) are followed by negative affect reduction and positive affect enhancement after the occurrence of the behavior. These findings have been replicated across different types of eating disorders including BN, anorexia nervosa, and binge eating in obesity [11,35,36]. These data support the premise that reward processes (i.e., negative and positive reinforcement through affect alteration) play a critical role in the maintenance of BN. However, previous EMA studies have not examined the impact of the anticipation of binge eating and purging on the trajectories of negative affect and positive affect in the natural environment.

### Frontostriatal Connectivity Patterns Are Related to Symptoms in Binge-Eating Samples

We recently examined the resting state functional connectivity of reward circuitry in 27 adults with binge-eating disorder compared with 21 control participants [37]. Compared to the control participants, the binge eating disorder group showed lower resting state functional connectivity in the striatal regions, with frontal regions (superior frontal gyrus) mediating executive processes and posterior, parietal, and temporal regions associated with emotional processing.

### Summary

Our preliminary data indicate that abnormalities in affect-based reward processes, as well as underlying disturbances in associated neurobiology (e.g., approach and threat circuits), contribute to the perpetuation of binge-eating and purging behaviors in BN. Our data also underscore that anticipation, independent of the behavior itself, may be rewarding in BN. Taken together, these results suggest that the neurobiologically-driven processes related to reward anticipation in addition to reward receipt may be critical to BN maintenance.

## RESEARCH APPROACH

### Participants

60 participants (30 with BN, 30 sex-, age-, and ethnicity-matched individuals without BN) will be recruited through online and community and clinic advertising. Specifically, recruitment flyers will be placed in local eating disorder treatment program locations, on college campuses, and throughout the local community. In addition, advertisements will be placed in mainstream and on-line advertisements, including social media. *Inclusion criteria*: All participants: age 18–55; right-handed. BN group: DSM-5 diagnosis of BN [38], defined as at least one binge eating and one self-induced vomiting per week for the past three months; binge-eating episodes are always accompanied by self-induced vomiting episodes; stable dose (at least six weeks) of any medications impacting mood, appetite, or weight (for example, stimulants, birth control pills, antidepressant medications). Non-BN comparison group: no current or past history of an eating disorder; no current medication impacting mood, appetite, or weight. *Exclusion criteria*: history of gastric bypass surgery; medical condition acutely affecting eating behavior and/or weight including pregnancy, lactation, thyroid disease); current medical or psychiatric instability (e.g., hospitalization required in the past three months, acute suicidality); lifetime history of psychosis or bipolar disorder; lifetime history of neurological disorder or head injury with greater than 10 minutes loss of consciousness; inability to read English; current substance use disorder; body mass index less than 19 kg/m^2^; MRI incompatibility (e.g.,braces,implantedmedicaldevice, inability/unwillingness to complete scanning); food allergy that cannot be accommodated through substitutions to the laboratory test snack. Participants with current mood, anxiety, personality, and attentional disorders will be included. Because these psychiatric comorbidities are common among individuals with BN, excluding individuals with these diagnoses would limit the generalizability of the study findings.

### Methods, Measures, and Procedures

**Figure 1.**
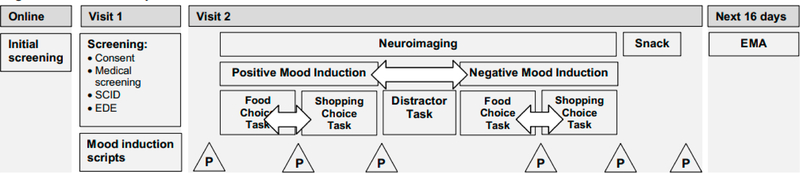
Overview of Study Procedures. Note: EDE = Eating Disorder Examination; P = Positive and Negative Affect Schedule (PANAS) questionnaire; SCID = Structured Clinical Interview for DSM-5. Arrows represent procedures that will be counterbalanced in a randomized order across participants.

Study procedures are outlined in Figure 1.

#### Visit 1.

Following informed consent procedures, participants with BN will complete the Eating Disorder Examination (EDE) [ 39], a semi-structured diagnostic and eating disorder assessment measure, with a trained clinical interviewer to confirm BN diagnosis as well as to determine that binge-eating episodes are consistently accompanied by self-induced vomiting. Recruiting individuals with this pattern of BN symptoms will reduce potential heterogeneity (e.g., by excluding individuals who sometimes purge without binge eating or vice versa) and will enable us to examine binge eating and purging as a “unit” rather than independently. The EDE will be used to rule out current and past eating disorders among potential non-BN participants and to determine duration of illness among BN participants. In addition, the Structured Clinical Interview for DSM-5 (SCID-5; [40]) will be administered to characterize cooccurring psychopathology including mood, anxiety, and substance-use disorders as well as trauma and PTSD symptoms, and to identify any exclusionary psychiatric disorders. Height and weight will be measured using a digital scale and a stadiometer. Participants will generate a positive and negative mood induction script [41]. For negative mood induction, participants will be asked to provide several examples of times when they “felt badly” about themselves, along with a rating (1–10) of how badly they felt They will then be asked to write a detailed description of the event when they felt most badly by re-imagining the experience and remembering the details as vividly as possible [41]. Similar procedures will be followed for the positive mood induction script. In addition, several self-report assessment questionnaires will be administered for sample description and secondary analyses including measures of emotion regulation (Difficulties in Emotion Regulation Scale [42]; UPPS-P Impulsive Behavior Scale [43]), clinical impairment (Clinical Impairment Assessment [44]), behavioral inhibition (Behavioral Inhibition System/Behavioral Activation system [45]), and current psychopathology (Beck Depression Inventory [46], State Trait Anxiety Inventory [47]).

#### Visit 2.

Participants will be scheduled for early morning appointments and will be asked to refrain from eating overnight prior to scanning and to abstain from alcohol, drugs, nicotine, and caffeine for 24 h prior to the second visit. Prior to scanning, participants will provide mood ratings on the Positive Affect Negative Affect Schedule (PANAS) [48] and practice versions of the in-scanner tasks to familiarize themselves with the procedures. During the fMRI, participants will undergo positive and negative mood induction (counterbalanced) and will complete two tasks (food choice and shopping choice tasks, also counterbalanced) in each condition, resulting in a 2 (group) × 2 (food versus shopping task) design that will be analyzed separately for positive and negative mood conditions. During the mood induction, participants will be instructed to vividly reimagine themselves in the situation that they had written about during Visit 1 (i.e., positive or negative, depending on the condition) while they listen to the researcher reading their script. Using the PANAS, they will rate mood before and after each mood induction, as well as after each task. In between the negative and positive mood conditions, participants will complete a distractor task that involves counting backward in increments of 3 for 60 s to neutralize mood. After scanning procedures, participants will be provided an opportunity to consume a snack selected during the food choice task in privacy and will receive an item viewed during the shopping choice task. Participants will rate mood on the PANAS before and after eating and receiving the item from the shopping task. Participants will then complete a standardized relaxation procedure to reduce any residual negative mood [18]. Participants will receive $100 after the completion of the second visit.

#### fMRI Behavioral Tasks.

To assess the neural correlates of binge-eating and purging anticipation, participants will complete an established food choice task [49] paired with loss-of-control eating instructions while in the scanner. In this task, participants provide ratings indicating preference for consuming one of two presented foods ranging in caloric density and percentage from fat, including “high-fat” foods (>30% of calories from fat) that are typical of those consumed during binge-eating episodes. The 8-minute task involves 76 food choices. As in prior investigations, participants will be told that their food selections will determine which food they will be given at a subsequent snack following scanning [49,50]. However, to avoid participants engaging in restrictive food choices, the instructions will be modified, asking participants to “let yourself go and eat as much as you want” during the snack. These instructions have been used in established feeding lab procedures to elicit loss of control binge-eating behavior [50] in individuals with and without eating disorders. During the snack, participants will be provided with a large portion of one of their highly-rated foods from the food choice task. They will eat in private and have access to a private, unmonitored room/bathroom so that they have the opportunity to binge eat (and, for participants with BN, purge), if desired. They will then be asked to rate (on a scale of 0–5) the extent to which they believed that they had engaged in a binge-eating episode, overeaten, and experienced loss of control. During a comparison shopping task, participants will provide ratings indicating their preference for one of two non-food items $20 in value (e.g., home décor items). The 8-minute task involves 76 item choices. After the scan and snack, they will be provided with an item that they had rated positively during the fMRI shopping task to take home.

### Neuroimaging Procedures

Scanning will be conducted with a 3 Tesla Siemens Prisma scanner at the Center for Magnetic Resonance Research at the University of Minnesota. A 32-channel receive only head coil will be used to obtain images for all participants. High-resolution anatomical images will be collected for each participant using a T_1_-weighted sequence (multi-echo MPRAGE: 208 sagittal slices, TR = 2500 ms, TE = 1.81, 3.60, 5.39, 7.18 ms, TI = 1000 ms, flip angle = 8°; FOV = 256 × 240 mm; voxel size = 0.8 × 0.8 × 0.8 mm isotropic; matrix size = 320 × 300) and T_2_-weighted sequence (SPACE: 208 sagittal slices, TR = 3200 ms, TE = 564 ms, variable flip angle, FOV = 256 × 240 mm, voxel size = 0.8 × 0.8 × 0.8 mm isotropic; matrix size = 320 × 300). During the fMRI tasks, functional data will be acquired using the Human Connectome Project multiband echo planar imaging sequence (MB-fMRI) where 485 T_2_*-weighted whole brain functional volumes (72 contiguous slices; TR = 800 ms, TE = 37 ms, flip angle = 55°, FOV = 208 × 208 mm; voxel size = 2 × 2 × 2 mm; matrix = 104 × 104; multiband factor = 8, 8 min) will be collected for each task.

### Imaging Data Pre-Processing

Structural T_1_- and T_2_- weighted data will be pre-processed using the Human Connectome Project (HCP) structural minimum preprocessing stream which includes anatomical parcellation using FreeSurfer [51]. fMRI data will be pre-processed using the HCP fMRI minimum preprocessing stream. Volumes exceeding 0.5 mm of frame-wise displacement from the previous volume will be removed using a confound matrix.

### fMRI Activation Analysis

For each subject, fixed-effects analyses will be conducted to model food choice and shopping using a canonical double-gamma hemodynamic response function with a temporal derivative and local autocorrelation correction. Implicit baseline trials will be left un-modeled to avoid over-specification and incorrectly biasing percent signal change estimates. We will extract average *z* scores from regions of interest (using FreeSurfer-defined masks for bilateral amygdala, nucleus accumbens, caudate, putamen, insula, anterior cingulate cortex, ventromedial prefrontal cortex, and dorsolateral prefrontal cortex) from the food choice versus shopping contrast for subsequent analyses.

### Ecological Momentary Assessment (EMA)

To assess momentary variables, participants with BN will begin 16 days of cell phone-administered EMA immediately following their fMRI visit. Participants will complete two days of “practice” EMA ratings (that will not be used in the analyses) in order to improve data quality and to reduce potential reactivity. EMA data will be collected using the ReTAINE® (Real Time Assessment in the Natural Environment) data management system and will include three types of daily self-report recordings [52]: (a) interval contingent, in which participants complete EMA ratings at designated time intervals; (b) signal contingent, in which participants respond to signals from their EMA device (i.e., participants will be signaled at random five times during the day and evening), and (c) event contingent, in which participants provide EMA recordings in response to a specific event (i.e., before and after eating, binge-eating and purging anticipation event). EMA signal contingent measures include ratings of positive and negative affect using the PANAS [48] and Profile of Mood States [53], ratings of overeating and loss of control based on EDE items [39] to determine binge-eating and vomiting frequency, and questions related to momentary binge-eating and purging anticipation (e.g., To what extent are you thinking about whether or not to binge and purge?” “To what extent are you planning a binge-eating and purging episode right now?” “To what extent are you planning specific foods to eat in a binge?” (ratings (1–5)). In addition to the $100 they will receive for completing scanning procedures, BN participants will receive $100 after EMA with an additional $50 bonus if they respond to 80% or more of the EMA signals.

### Statistical Analyses

Hypothesis testing will be conducted using multilevel mixed-effects models based upon a general linear or generalized linear model, depending upon distributional requirements. Analyses will be based upon all available data and missing data will not be imputed. Covariates in hypothesis testing will include sex and age.

#### Hypothesis 1.1.

We hypothesize that following negative mood induction, participants with BN will show lower levels of self-reported negative affect, lower levels of activation in regions of the limbic threat network (e.g., amygdala, insula), and greater frontolimbic connectivity during an fMRI food choice task compared to the shopping task. We hypothesize that in contrast, healthy comparison participants will show no differentiation in these responses between tasks after a negative mood induction.

A mixed-effects model based upon a general or generalized linear (depending upon distribution of outcome) will be used to compare the effects of group and task (food choice vs shopping task) on self-reported negative affect (PANAS), activation within threat circuit regions of interest, and frontolimbic connectivity in the negative mood induction. Models will include a random intercept, and fixed effects for group, task, and group-by-task interaction, with the test of the hypothesis based upon the interaction. The pre-task affect assessment will be included as a covariate in all affect models.

#### Hypothesis 1.2.

We hypothesize that following positive mood induction, participants with BN will show higher levels of self-reported positive affect, higher levels of activation in regions of the striatal approach network (e.g., nucleus accumbens, caudate), and lower frontostriatal connectivity during an fMRI food choice task compared to the shopping task. We hypothesize that in contrast, healthy comparison participants will show no differentiation in these responses between tasks after positive mood induction.

A mixed-effects model will be used to compare the effects of group and task (food choice vs shopping task) on self-reported positive affect (PANAS) and activation in approach circuit regions of interest, and frontostriatal connectivity in the positive mood induction. Models will include a random intercept, and fixed effects for group, task, and group-by-task interaction, with the test of the hypothesis based upon the interaction.

#### Hypothesis 1.3.

We hypothesize that for participants with BN, illness duration will moderate responses to an fMRI food choice task. Specifically, we predict that longer illness duration will be associated with: (a) less negative affect, less activation of the limbic threat network, and more frontolimbic connectivity following the negative mood induction; and (b) more positive affect, more activation of striatal approach regions, and less frontostriatal connectivity following the positive mood induction.

Pearson correlations will be calculated to examine the relationship between BN duration, self-reported affect (PANAS), neural activation in threat and approach circuit, regions of interest, and frontolimbic and frontostriatal connectivity in the BN food choice condition separately for each mood state.

#### Hypothesis 2.1.

We hypothesize that the magnitude of EMA negative affect reduction with binge-eating and purging behavior among individuals with BN will be associated with the magnitude of limbic threat network activity and frontolimbic connectivity observed during an fMRI food choice task in the negative mood induction condition.

The average magnitude of negative affect pre-to-post change with binge-eating and purging anticipation will be calculated separately for each individual from EMA data. Pearson correlations will be calculated to examine the relationship between the average magnitude of negative affect reduction following binge-eating and purging anticipation as measured by EMA with the magnitude of activity in threat circuit regions of interest and frontolimbic connectivity during the fMRI food choice task in the negative mood condition.

#### Hypothesis 2.2.

We hypothesize that the magnitude of EMA positive affect increase with binge-eating and purging behavior among individuals with BN will be associated with the magnitude of striatal approach network activity and frontostriatal connectivity during an fMRI food choice task in the positive mood induction condition.

Similar procedures as those described for Hypothesis 2.1 will be used to examine the association between positive affect and activity in approach circuitry regions of interest and frontostriatal connectivity during the fMRI food choice task in the positive mood condition.

#### Hypothesis 2.3.

We hypothesize that greater EMA binge-eating and purging frequency will correlate with greater magnitude of reductions in negative affect and increases in positive affect associated with binge-eating and purging anticipation as measured by EMA. We also hypothesize that greater EMA binge-eating and purging frequency will correlate with fMRI measures of limbic threat and striatal approach network activity, as well as connectivity among individuals with BN.

Frequencies of binge-eating and purging episodes per week will be calculated for each individual from EMA data. Pearson correlations will be calculated between the frequency of binge-eating and purging behavior as measured by EMA, the magnitude of changes in mood as measured by EMA, and frontolimbic and frontostriatal activity/connectivity.

### Power Analysis

Effect size estimates for fMRI comparisons between the BN and non-BN groups are based upon earlier findings [18] in which the BN group showed greater deactivation across the bilateral amygdala and greater activation averaged across the bilateral caudate during a binge planning task compared to a furniture selection task, with large effect sizes (*d* > 1.0) observed for these differences. While estimates for negative and positive affect differences between groups are not available, we expect these differences to be much smaller, perhaps in the small-to-medium effect (*d* = 0.40–0.50) range. *Hypotheses 1.1–1.2*: The proposed sample size of 60 participants (30 BN, 30 HC) provides a power of 0.87 to detect a group × task interaction with an effect size (*d*) of 0.40 and a power of 0.97 to detect an effect size of 0.51. Thus, power sh ould be adequate for affect comparisons and excellent for activity/connection comparisons. Hypothesis 1.3: The proposed sample size of 30 BN participants provides a power of .78 to detect a mood induction-by-BN duration interaction with an effect size of 0.51 and a power of 0.86 to detect an effect size of 0.58. Hypotheses 2.1–2.3: The proposed sample size of 30 BN participants provides a power of 0.81 to detect a bivariate Pearson correlation of 0.50.

## SUMMARY AND FUTURE DIRECTIONS

This proposed study seeks to establish the psychological and neurobiological mechanisms associated with anticipatory reward in BN to support the importance of these processes in the maintenance of BN psychopathology. The specific aims of this study are to examine the impact of anticipatory reward and planning in BN on psychological, affective, and neurobiological reward indices. Specifically, the results of this study will demonstrate the neurobiological correlates of reward anticipation in the context of positive and negative emotions as well as the associations between these neurobiological processes and bulimic behavior in the natural environment. The study findings will also determine the extent to which duration of illness is associated with the magnitude of these psychological and neurobiological processes. Study findings will potentially inform novel treatment targets of anticipated and “planned” BN behaviors in existing and novel interventions, particularly the possibility that more distal anticipatory processes can be targeted in addition to more immediate behavioral precipitants. Subsequent applications will determine the extent to which these anticipatory reward processes occur in the varying levels of negative and positive affect, the role of habit and duration of illness and the potential distinctions between the anticipation of binge eating compared to purging.

## ABBREVIATIONS

BN: bulimia nervosa
CBT: cognitive behavioral therapy
EMA: ecological momentary assessment
fMRI: functional magnetic resonance imaging
EDE: Eating Disorder Examination
SCID-5: Structured Clinical Interview for DSM-5
PANAS: Positive and Negative Affect Schedule
